# Allele-aware chromosome-level genome assembly of the autohexaploid *Diospyros kaki* Thunb

**DOI:** 10.1038/s41597-023-02175-2

**Published:** 2023-05-11

**Authors:** Huawei Li, Peng Sun, Yiru Wang, Zhongren Zhang, Jun Yang, Yujing Suo, Weijuan Han, Songfeng Diao, Fangdong Li, Jianmin Fu

**Affiliations:** 1 Research Institute of Non-timber Forestry, Chinese Academy of Forestry, No. 3 Weiwu Road, Jinshui District, Zhengzhou 450003 China; 2 Key Laboratory of Non-timber Forest Germplasm Enhancement & Utilization of State Administration of Forestry and Grassland, No. 3 Weiwu Road, Jinshui District, Zhengzhou 450003 China; 3 Key Laboratory of Cultivation and Protection for Non-Wood Forest Trees, Ministry of Education, Central South University of Forestry and Technology, No. 498 Shaoshan South Road, Changsha, 410004 China; 4 Novogene Bioinformatics Institute, Beijing, 100083 China; 5 Shanghai Key Laboratory of Plant Functional Genomics and Resources, Shanghai Chenshan Plant Science Research Center, Chinese Academy of Sciences, Shanghai Chenshan Botanical Garden, 3888 Chenhua Road, Shanghai, 201602 China; 6 Henan Key Laboratory of Germplasm Innovation and Utilization of Eco-economic Woody Plant, Pingdingshan University, Pingdingshan, 467000 China

**Keywords:** Genome duplication, Genome

## Abstract

Artificially improving persimmon (*Diospyros kaki* Thunb.), one of the most important fruit trees, remains challenging owing to the lack of reference genomes. In this study, we generated an allele-aware chromosome-level genome assembly for the autohexaploid persimmon ‘Xiaoguotianshi’ (Chinese-PCNA type) using PacBio CCS and Hi-C technology. The final assembly contained 4.52 Gb, with a contig N50 value of 5.28 Mb and scaffold N50 value of 44.01 Mb, of which 4.06 Gb (89.87%) of the assembly were anchored onto 90 chromosome-level pseudomolecules comprising 15 homologous groups with 6 allelic chromosomes in each. A total of 153,288 protein-coding genes were predicted, of which 98.60% were functionally annotated. Repetitive sequences accounted for 64.02% of the genome; and 110,480 rRNAs, 12,297 tRNAs, 1,483 miRNAs, and 3,510 snRNA genes were also identified. This genome assembly fills the knowledge gap in the autohexaploid persimmon genome, which is conducive in the study on the regulatory mechanisms underlying the major economically advantageous traits of persimmons and promoting breeding programs.

## Background & Summary

Persimmon (*Diospyros kaki* Thunb.), a member of the Ebenaceae, is an important fruit tree species that originated in East Asia and was successively introduced to Europe and America in the 18th–20th centuries^1^. The cultivated area of persimmon had reached 1.01 million ha, with a total yield of 4.24 million tons globally in 2020 (www.fao.org). The persimmon industry is negatively affected by labor-intensive artificial de-astringency treatment, transportation difficulties, short shelf life, and limited processing^2^.

To enhance the persimmon industry, superior cultivars suitable for fresh-eating and processing are urgently needed. Current persimmon cultivars are generally classified into either pollination-constant non-astringent (PCNA) or non-PCNA^3,4^, based on the natural de-astringency capacity of fruits at the commercial maturity stage (fruits with mature peel color but not soft). The PCNA contains the Japanese-type PCNA (JPCNA) and Chinese-type PCNA (CPCNA), which are edible without any artificial de-astringency treatment and have high market valuable^5^. The non-PCNA includes pollination-variant non-astringent (PVNA), pollination-constant astringent (PCA), and pollination-variant astringent (PVA)^1^.

In the PCNA breeding program, inbreeding repression limits the efficiency due to the high genetic similarity among the JPCNA cultivars^6,7^. Modern molecular refinement breeding methods, including CRISPR/Cas9, are potentially effective for breeding new superior cultivars. The molecular mechanisms regulating crucial traits should first be determined accurately when using these new methods. The natural de-astringency capacity of JPCNA is controlled by a recessive allele at a single locus of ASTRINGENCY (*AST*)^8–10^. A previous study suggested that the natural de-astringency capacity of CPCNA is controlled by dominant alleles^11,12^, indicating that the CPCNA type may be more effective for breeding new superior PCNA cultivars, although the precise natural de-astringency mechanism of CPCNAs remains unknown.

The most common persimmon cultivar is hexaploidy (2n = 6x = 90). Owing to a lack in genomic data, the principal molecular mechanisms underlying the natural de-astringency of CPCNA and other crucial traits, including fruit size, shape, and flavor, of hexaploidy persimmon remains challenging to understand. Fortunately, the publication of genomes of hexaploid persimmon close relatives *Diospyros lotus* (2n = 2x = 30) and *Diospyros oleifera* (2n = 2x = 30) provided help for the study of persimmon biology^13–16^. Hexaploid and diploid persimmons are different species with discrepant genomic information. Taking the diploid persimmon genome as a reference, data on the regulation mechanism of some traits in hexaploid persimmon are limited, which contributes to the reduction of breeding efficiency; hence, the desperate need for the hexaploid persimmon genome assembly, that will help in both basic and applied research.

The assembly of polyploid genomes is a major technical challenge hindered by repeat content, transposable elements, high heterozygosity, and gene content^17^. The assembly of autopolyploids with smaller genetic distances is more susceptible to the misassignment of sub-genome fragments than allopolyploids. With the advancement of sequencing and assembly technology, the autopolyploid genomes of some plants have been reported, such as *Ipomoea batatas*^18^, *Saccharum spontaneum*^19,20^, *Medicago sativa*^21^, and *Solanum tuberosum*^22^, which provide a reference for current genome assemblies.

This study uses PacBio circular consensus sequencing (CCS) and high-throughput chromosome conformation capture (Hi-C) technologies to generate an allele-aware chromosome-level genome assembly for *D. kaki*. The current genomic information will provide a molecular platform for future research and elaborate breeding programs.

## Methods

### Sampling and sequencing

‘Xiaoguotianshi’ persimmon is one of the five varieties of the CPCNA persimmon ‘Luotiantianshi’ with a good taste and higher soluble solids content than other CPCNA persimmons. The young leaves of *D. kaki* ‘Xiaoguotianshi’ and *D. lotus* (wild germplasm) were collected from the Persimmon Germplasm Resources Nursery of Research Institute of Non-timber Forestry, Chinese Academy of Forestry (Yuanyang County, Henan Province, China, 34°55′18″–34 °56′27″N, 113°46′14″–113°47′35″E).

Genomic DNA was extracted from the young leaf tissue of *D. kaki* using a DNAsecure Plant Kit (TIANGEN, Beijing, China). Sequencing libraries with insert sizes of 350 bp were constructed using a library construction kit, following manufacturer’s instructions (Illumina, San Diego, CA, USA). The libraries were sequenced using the Illumina HiSeq X platform.

For the PacBio library, the DNA was used to construct 15-kb-insert-size SMRTbell libraries using the SMRTbell Express Template Prep Kit 2.0, following manufacturer’s instructions (PacBio, CA). Then, libraries were sequenced using PacBio Sequel II, and HiFi reads were obtained using the CCS tool (https://github.com/PacificBiosciences/ccs; v6.0.0) by setting ‘min-passes = 3, min-rq = 0.99’.

For the Hi-C library, formaldehyde was used to fix the chromatin. Leaf cells were lysed, and HindIII endonuclease was used to digest the fixed chromatin. The 5 overhangs of the DNA were recovered with biotin-labeled nucleotides, and the resulting blunt ends were ligated to each other using DNA ligase. Proteins were removed with protease to release DNA molecules from the crosslinks. The purified DNA was sheared into 350-bp fragments and ligated to adaptors^23^. The biotin-labeled fragments were extracted using streptavidin beads; following PCR enrichment, the libraries were sequenced on an Illumina HiSeq X instrument.

For RNA sequencing, total RNA was extracted from the leaf, stem and fruit tissues using an RNAprep Pure Plant Kit (TIANGEN, Beijing, China), and genomic DNA contaminants were removed using RNase-Free DNase I (TIANGEN, Beijing, China). The RNA integrity was evaluated using 1.0% agarose gel stained with ethidium bromide (EB), while its quality and quantity were assessed using an Agilent 2100 Bioanalyzer (Agilent Technologies, CA, USA). The integrated RNA was then used for cDNA library construction, Illumina and PacBio sequencing. The cDNA libraries were constructed using the NEBNext Ultra RNA Library Prep Kit (NEB, MA, USA) for Illumina and SMRTbell Express Template Prep Kit 2.0 (PacBio, CA, USA) for PacBio, following the manufacturers’ instructions. Prepared libraries were sequenced on the Illumina HiSeq X and PacBio Sequel platform.

### Genome size estimation

**Fig. 1:**
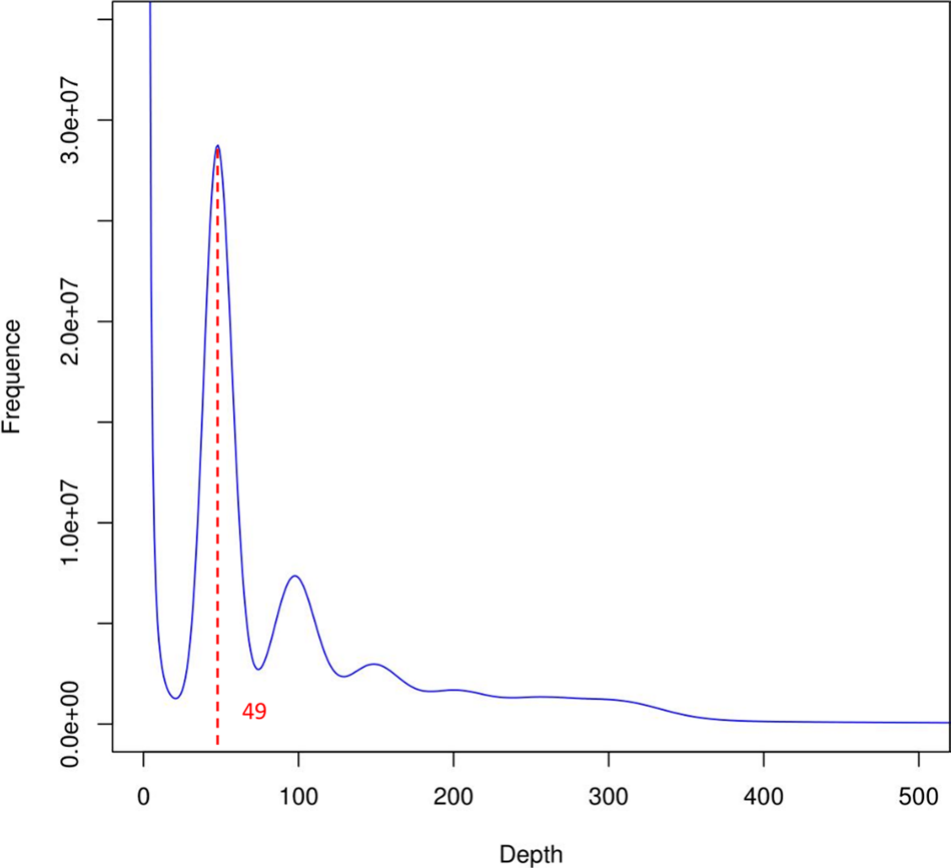
Results of 27-mer frequency analysis to estimate the *D. kaki* genome size. The haplotype genome size was calculated by dividing the total K-mer count by coverage-depth (222,144,314,592 /49 = 4,533,557,441).

K-mer frequency analysis was used to determine genome characteristics^24^. The genome size of *D. kaki* was calculated based on k-mer (k = 27) statistics using the modified Lander–Waterman algorithm. The total length of the sequence reads was divided by the sequencing depth; the peak value of the frequency curve represented the overall sequencing depth. We estimated the genome size using the following formula: (N × (L−K + 1) − B)/D = G, where N is the total number of the sequence reads, L is the average length of the sequence reads, K is the K-mer length (27 bp)^25^, B is the total number of low-frequency K-mers (frequency ≤ 1 in this analysis), G is the genome size, and D is the overall depth estimated via the K-mer distribution. Heterozygosity was reflected in the distribution of the number of distinct k-mers (k = 27). On the basis of a total of 222,144,314,592 27-mer and a peak 27-mer depth of 49, the estimated genome size was 4533.56 Mb (Fig. 1).

The genome size of the sequenced individuals was confirmed using flow cytometry. Approximately 20–50 mg of fresh leaves of *D. kaki* and *D. lotus* were chopped using a razor blade in 1 ml of LB01 buffer (15 mM Tris, 2 mM Na2EDTA, 0.5 mM spermine tetrahydrochloride, 80 mM KCl, 20 mM NaCl, 0.1% (vol/vol) Triton X-100) adjusted to pH 7.5 with 1 M NaOH and b-mercaptoethanol to 15 mM. Cell culture was collected by gentle pipetting and filtered through a 400-mesh nylon strainer. The samples were stained with 100 μg/ml PI and 100 μg/ml RNase in an ice bath for 10 min before analysis using a MoFlo-XDP flow cytometer (Beckman Coulter Inc., USA).

**Fig. 2:**
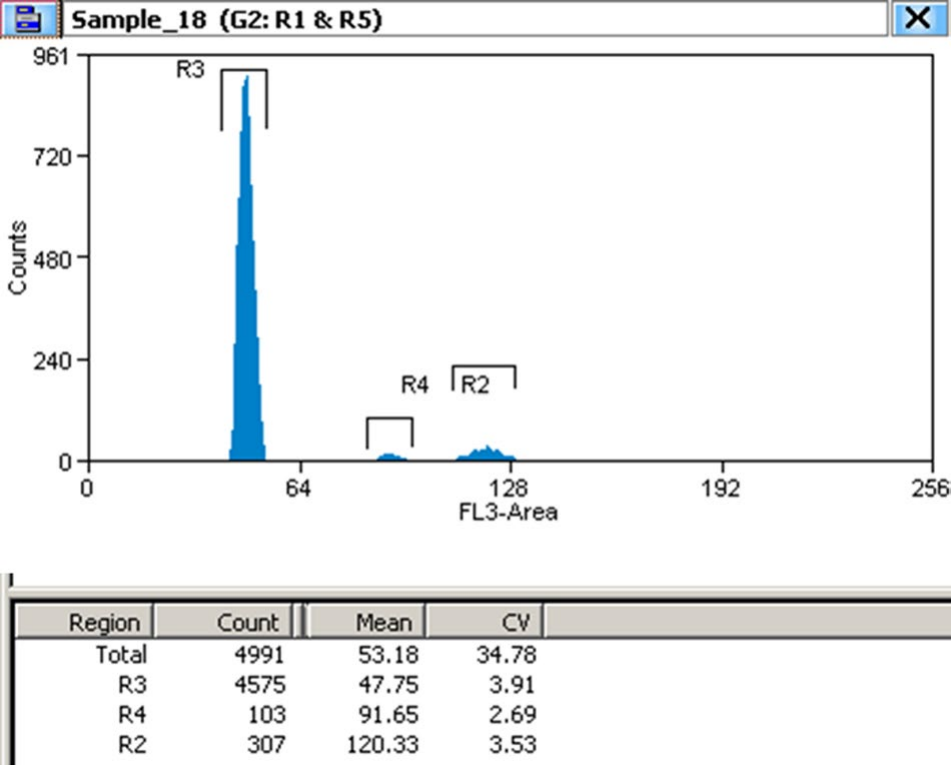
Results of flow cytometry analysis to estimate the *D. kaki* genome size. The *D. lotus* genome (2n = 2x = ~1.76 Gb) served as an internal reference standard. Peak R3 showed DNA amount of *D. lotus*. Peak R2 showed duplicated DNA amount of *D. lotus*. The ratio of peak mean was equal 2.52 (R2/R3) and 1.31 (R2/R4), hence the estimated genome size of *D. kaki* was 2n = 6x = ~4.44 Gb and 4.61 Gb. Due to the distance between peak R2 and peak R4 is less than the distance between peak R2 and peak R3, it is more accurate to estimate genome size of *D. kaki* 2n = 6x = ~4.61 Gb.

Nuclear fluorescence was measured using a MoFlo-XDP high-speed flow cytometer with a 70 μm ceramic nozzle at a sheath pressure of 60 psi. PI fluorescence was detected with a solid-state laser (488 nm) and a 625-/26-nm HQ band-pass filter. The FL3-Height/SSC-Height gate method eliminated debris, cell fragments, and dead cells. Single and double cells were discriminated using FL3-Height /FL3-Area. The final results showed that the genome size of *D. kaki* was 4.61 Gb (Fig. 2).

### Genome assembly

**Fig. 3:**
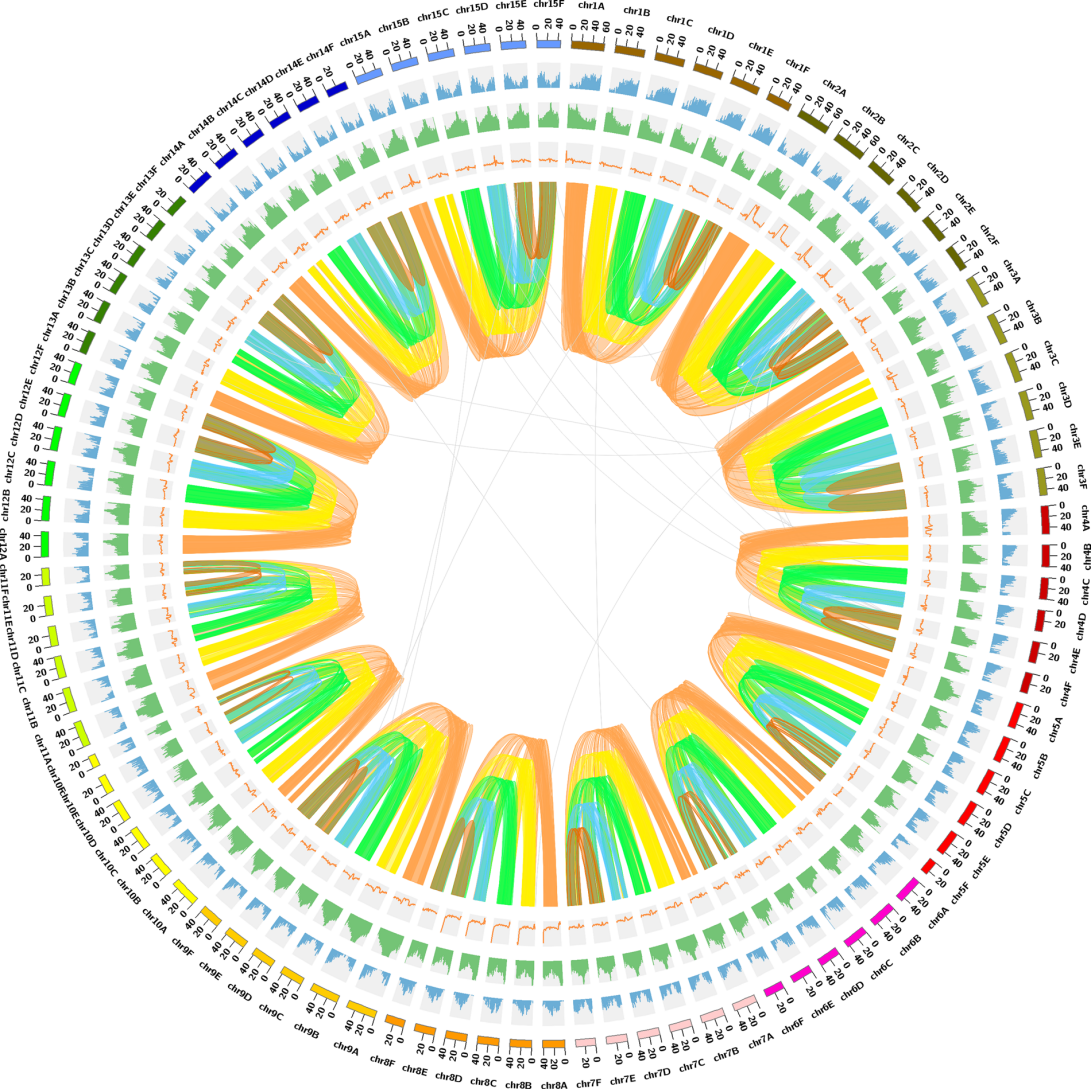
Overview of the *D. kaki* genome. From the outer ring to the inner ring are Chromosome, Gene density, TE density, GC content, and Synteny.

**Fig. 4:**
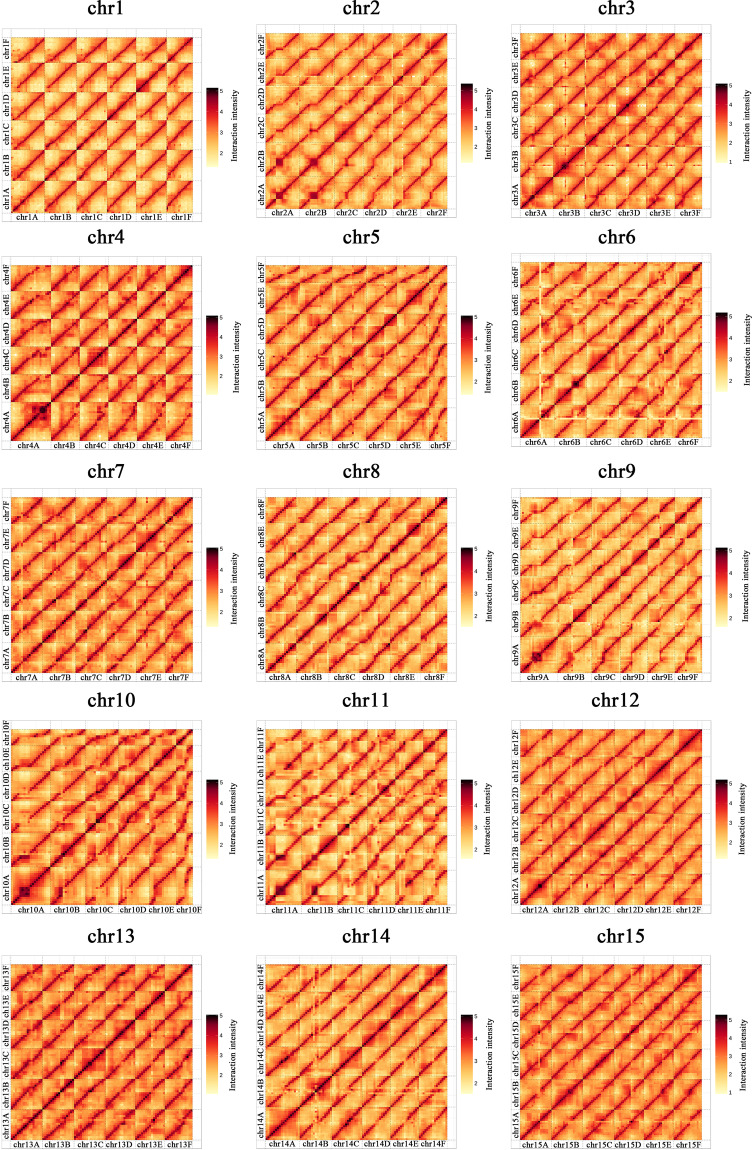
Overview of Hi-C contacts in the heat map visualization for assembled chromosomes.

In total, 179.09 Gb PacBio HiFi long reads (8 SMRT cell; 39.53X coverage) and 445.72 Gb Hi-C paired-end reads (98.39X coverage) were obtained (Table 1). *D. kaki* genome was assembled with Hifisam (v0.13-r308)^26^ using PacBio HiFi reads with default parameter settings. After initial assembly, Hi-C sequencing data were aligned to the assembled contigs using the Burrows-Wheeler Aligner (BWA) mem option^27^, while pseudo-chromosomes were constructed based on ALLHIC (v0.9.8)^20^. We configured the parameter setting -K 90--minREs 50--maxlinkdensity 3--NonInformativeRabio 2. Finally, Hi-C scaffolding yielded 90 chromosome-length scaffolds. The final assembly contained 4.52 Gb with a contig N50 value of 5.28 Mb and scaffold N50 value of 44.01 Mb, respectively; 4.06 Gb (89.87%) of the assembly was anchored onto 90 chromosome-level pseudomolecules comprising 15 homologous groups, with six allelic chromosomes in each. The assignment to genome haplotypes was based on chromosome length (Tables 2, 3; Figs. 3, 4).

**Table 1:** Statistics of data for genome assemblies of *D.kaki*.

Read type	Read base (Gb)	Number of reads	Mean read length (bp)	Read length (N50)
HiFi reads	179.09	12,077,194	14,828	14,875
Hi-C reads	445.72	1,485,733,937	150	—

**Table 2:** Summary of *D. kaki* genome assembly.

Genome assembly	Number	Size
Total contigs	22,172	4.52 Gb
Contig N50	220	5.28 Mb
Contig N90	6,674	38.41 Kb
Total scaffolds	12,715	4.52 Gb
Scaffold N50	45	44.01 Mb
Scaffold N90	97	751.00 Kb
Pseudo-chromosomes	90	4.06 Gb

**Table 3:** Statistics of chromosome length in *D. kaki* genome.

Chromosome	DkaA	DkaB	DkaC	DkaD	DkaE	DkaF
	Length (bp)	Length (bp)	Length (bp)	Length (bp)	Length (bp)	Length (bp)
chr1	61,854,060	56,088,851	55,416,202	53,982,736	53,941,820	46,325,742
chr2	61,828,268	61,482,766	53,378,268	51,130,973	51,039,378	47,917,465
chr3	59,673,878	58,299,181	56,577,420	55,427,238	52,248,968	50,631,600
chr4	52,914,409	40,854,466	40,785,913	39,946,333	39,656,787	38,564,113
chr5	48,795,537	48,609,808	48,061,808	46,857,559	45,974,615	27,067,684
chr6	47,859,392	46,520,457	44,013,165	40,127,273	39,913,631	36,538,425
chr7	44,533,068	43,838,789	41,532,886	41,183,422	39,021,685	37,333,795
chr8	42,161,358	40,923,269	40,632,031	40,148,576	39,064,181	36,013,403
chr9	56,056,215	54,404,229	43,939,775	42,977,330	42,624,317	41,332,283
chr10	51,658,814	43,541,839	42,887,684	40,037,245	34,419,546	23,313,593
chr11	47,898,209	46,534,985	41,284,961	37,036,693	36,825,070	32,340,415
chr12	47,594,529	46,266,353	46,003,302	45,360,257	43,649,862	41,231,788
chr13	43,497,036	42,187,849	40,963,846	40,947,171	40,100,542	38,578,397
chr14	46,638,359	45,764,966	40,779,368	40,279,559	40,271,275	39,884,115
chr15	50,207,220	50,127,302	49,236,925	48,601,615	46,818,767	44,634,995
Total	763,170,352	725,445,110	685,493,554	664,043,980	645,570,444	581,707,813

### Repetitive sequence annotation

Transposable elements (TEs) in the *D. kaki* genome were identified by combining *de novo-* and homology-based approaches. For the *de novo*-based approach, we used RepeatScout (v1.0.5; https://github.com/mmcco/RepeatScout)^28^, RepeatModeler (http://www.repeatmasker.org/RepeatModeler.html), and LTR_FINDER (v1.0.7; https://github.com/xzhub/LTR_Finder)^29^ to build a *de novo* repeat library. For the homology-based approach, we used RepeatMasker (v3.3.0; http://www.repeatmasker.org/) against the Repbase TE library (http://www.girinst.org/server/RepBase/)^30^ with score cut-off of 225 and RepeatProteinMask (v4.0.5; http://www.repeatmasker.org/) against the TE protein database^31^ with a p-value cut-off of 1e-4. Tandem repeats were identified using Tandem Repeats Finder (v4.0.7; https://tandem.bu.edu/trf/)^32^ with parameters ‘matching weight: 2, mismatching penalty: 7, indel penalty: 7, minimum alignment score:50, maximum period size: 2000’. Ultimately, a total of 2.90 Gb of repetitive elements occupying 64.02% of the *D. kaki* genome were annotated (Table 4). Most of the repeats were long terminal repeats (LTRs) (51.28% of the genome; Table 5). The DNA, LINE, and SINE classes accounted for 5.93%, 2.66%, and 0.03% of the genome, respectively (Table 5).

**Table 4:** Summary of repeat sequences in *D. kaki* genome.

Type	Length (bp)	Percent (%)
Tandem Repeat Finder	616,854,190	13.64
RepeatMasker	2,742,911,542	60.64
RepeatProteinMask	762,622,147	16.86
Total	2,896,122,867	64.02

**Table 5:** Summary of TE sequences in *D. kaki* genome.

Type	Length (bp)	Percent (%)
DNA	268,202,039	5.93
LINE	120,230,805	2.66
SINE	1,375,715	0.03
LTR	2,319,894,281	51.28
Unknown	121,044,880	2.68
Total	2,823,554,370	62.42

### Gene prediction and annotation

Homology-based, *de novo*, and transcriptome-based predictions were used to predict protein-coding genes in the *D. kaki* genome. Homologous proteins from five plant genomes (*Arabidopsis thaliana, D. oleifera, D. lotus, Actinidia chinensis*, and *Camellia sinensis*) were downloaded from Ensembl Plants (http://plants.ensembl.org/index.html) and NCBI (https://www.ncbi.nlm.nih.gov/). The protein sequences were then aligned to the *D. kaki* genome assembly using tblastN^33^, with an E-value cut-off of 1e-5. The BLAST hits were conjoined using a Solar software^34^. GeneWise (https://www.ebi.ac.uk/Tools/psa/genewise) was used to predict the exact gene structure of the corresponding genomic regions in each BLAST hit (Homo-set)^35^. The published RNA-seq data of female flowers and fruit at different developmental stages, and 0.33 Gb new sequencing RNA-seq data of the young leaves and stems of ‘Xiaoguotianshi’ (three biological replicates) were mapped to the *D. kaki* genome using HISAT2 (https://daehwankimlab.github.io/hisat2/, v2.2.1)^36^ and Cufflinks (http://cole-trapnell-lab.github.io/cufflinks/, v2.1.1)^37^ (Table 6). A total of 70.54 Gb Iso-seq data from PacBio transcriptome sequencing of mixed samples containing the young leaves, stems, and fruits of ‘Xiaoguotianshi’ (three biological replicates) were used to create several pseudo-ESTs. These pseudo-ESTs were mapped to the assembly, and gene models were predicted using PASA (http://pasapipeline.github.io/)^38^ (Table 6). This gene set was denoted as the PASA-T-set and used to train *ab initio* gene prediction programs. Five *ab initio* gene prediction programs, namely, Augustus (http://augustus.gobics.de/, v3.2.3), GENSCAN (http://genes.mit.edu/GENSCAN.html, v1.0), GlimmerHMM (http://ccb.jhu.edu/software/glimmerhmm/, v3.0.1), geneid (http://genome.crg.es/software/geneid/), and SNAP (http://korflab.ucdavis.edu/software.html) were used to predict coding regions in the repeat-masked genome^39–42^. Gene model evidence from homo-set, cufflinks-set, PASA-T-set, and *ab initio* programs were combined using EVidenceModeler (EVM) (http://evidencemodeler.sourceforge.net/) into a non-redundant set of gene structures^43^.

**Table 6:** Statistics of RNA-seq and Iso-seq.

Read type	Read base (Gb)	Number of reads	Mean read length (bp)	Read_length (N50)
RNA-seq	0.33	—	150	—
Iso-seq	70.54	715,846	98,537	166,494

Functional annotation of protein-coding genes was performed using BLASTP (E-value: 1e-05) against two integrated protein sequence databases^44^: SwissProt (http://web.expasy.org/docs/swiss-prot_guideline.html) and NR (ftp://ftp.ncbi.nih.gov/blast/db/). Protein domains were annotated by searching against the InterPro (http://www.ebi.ac.uk/interpro/, v32.0) and Pfam (https://pfam-legacy.xfam.org/.org/, v27.0) databases using InterProScan (v4.8) and HMMER (http://www.hmmer.org/, v3.1), respectively^45–48^. Gene ontology (GO, http://www.geneontology.org/page/go-database) terms for each gene were obtained from the corresponding InterPro or Pfam entries. The pathways in which the genes might be involved were assigned using BLAST against the Kyoto Encyclopedia of Genes and Genomes (KEGG) database (http://www.kegg.jp/kegg/kegg1.html, release 53), with an E-value cut-off of 1e-05. Overall, a total of 153,288 protein-coding genes were predicted with an average sequence length of 7,397.94 bp and an average CDS length of 1,153.82 bp (Table 7). Of these, 135,446 genes are anchored to 90 chromosomes (Table 8). On average, each predicted gene contained 5.01 exons with an average sequence length of 230.33 bp (Table 7). 98.60% of the genes were functionally annotated via similarity searches against homologous sequences and protein domains (Table 9).

**Table 7:** Summary of gene structure prediction in *D. kaki* genome.

Gene set		Number	Average gene length (bp)	Average CDS length (bp)	Average exons per gene	Average exon length (bp)	Average intron length (bp)
De novo	Augustus	177,974	5,346.69	1,022.08	4.14	246.89	1,377.34
	GlimmerHMM	321,260	10,295.63	601.95	3.09	194.89	4,641.03
	SNAP	319,132	7,138.48	610.14	3.33	183.25	2,802.42
	Geneid	390,239	3,850.38	577.88	3.24	178.29	1,460.13
	Genscan	230,229	10,666.94	976.81	5.22	187.04	2,294.9
Homolog	Dlo	359,850	1,914.73	816.37	2.76	296.06	624.97
	Ath	207,508	3,745.85	932.2	3.17	294.28	1,297.97
	Dol	227,166	4,359.26	895.42	3.6	248.67	1,331.78
	Ach	245,176	3,571.73	835.91	2.77	301.63	1,544.5
	Csi	145,462	5,893.18	1,125.04	4.23	266.16	1,477.65
RNA-seq	Cufflinks	239,070	11,977.18	2,111.23	6.58	320.70	1,767.08
	PASA	54,520	10,118.79	1,743.09	6.33	275.39	1,571.55
EVM		190,809	6,293.70	1,010.92	4.37	231.18	1,566.28
PASA-update		190,490	6,309.20	1,018.09	4.39	231.90	1,560.69
Final set		153,288	7,397.94	1,153.82	5.01	230.33	1,557.38

**Table 8:** Statistics of chromosome gene number in *D. kaki* genome.

Chromosome	DkaA	DkaB	DkaC	DkaD	DkaE	DkaF
	gene number	gene number	gene number	gene number	gene number	gene number
chr1	2,633	2,509	2,486	2,420	2,259	2,235
chr2	1,922	1,970	1,973	2,140	1,974	1,894
chr3	2,084	2,018	2,117	1,967	2,172	2,028
chr4	1,481	1,515	1,465	1,522	1,445	1,469
chr5	1,717	1,656	1,667	1,638	1,675	1,114
chr6	1,442	1,486	1,451	1,386	1,306	1,207
chr7	1,466	1,355	1,265	1,296	1,363	1,309
chr8	1,261	1,515	1,318	1,031	1,271	1,103
chr9	1,189	1,264	1,235	1,349	1,204	1,146
chr10	1,326	1,207	1,162	1,216	1,147	845
chr11	1,036	1,057	1,049	962	1,114	961
chr12	1,556	1,540	1,489	1,515	1,621	1,357
chr13	1,144	1,167	1,123	1,193	1,137	1,179
chr14	1,351	1,198	1,250	1,216	1,263	1,182
chr15	1,766	1,762	1,822	1,768	1,687	1,695
Total	23,374	23,219	22,872	22,619	22,638	20,724

**Table 9:** Statistics of gene function annotation in *D. kaki* genome.

Database		Number	Percent (%)
NR		134,846	88.0
Swiss-Prot		105,533	68.8
KEGG		100,175	65.4
InterPro	All	150,267	98.0
	Pfam	103,027	67.2
	GO	136,771	89.2
Annotated		151,088	98.6

tRNA genes were identified using the tRNAscan-SE software^49^. The rRNA fragments were predicted by aligning the rRNA sequences using BlastN at an E-value of 1e-10. The miRNA and snRNA genes were predicted using the INFERNAL software^50^ against the Rfam database (release 9.1)^51^. As a result, 110,480 rRNA, 12,297 tRNA, 1,483 miRNA, and 3,510 snRNA genes were annotated (Table 10).

**Table 10:** Statistics of non-coding RNA in *D. kaki* genome.

Type		Copy	Average length (bp)	Total length (bp)
miRNA		1,483	122.95	182,342
tRNA		12,297	75.59	929,561
rRNA	rRNA	110,480	234.59	25,917,998
	18 S	8,496	1544.7	13,123,748
	28 S	28,169	141.96	3,998,950
	5.8 S	7,158	161.1	1,153,155
	5 S	66,657	114.65	7,642,145
snRNA	snRNA	3,510	112.12	393,524
	CD-box	2,593	102.02	264,544
	HACA-box	237	132.64	31,435
	splicing	670	142.11	95,211

## Data Records

Raw data of genome sequencing and transcriptome sequencing of *D. kaki* are deposited in the NCBI SRA database under BioProject ID PRJNA810977. The SRA accession number of PacBio HiFi sequencing data are SRR18500470^52^, SRR18500471^53^, SRR18500472^54^, SRR18500473^55^ SRR18500474^56^, SRR18500475^57^, SRR18500476^58^, and SRR18500477^59^. The SRA accession number of Hi-C sequencing data are SRR18500481^60^, SRR18500482^61^, SRR18500483^62^, SRR18500484^63^, SRR18500485^64^, SRR18500486^65^, SRR18500487^66^ and SRR18500488^67^. The SRA accession number of Illumina sequencing data are SRR18500479^68^ and SRR18500480^69^. The SRA accession number of Iso-seq data SRA accession number is SRR18500463^70^. The SRA accession number of some RNA-seq data are SRR18500464^71^, SRR18500465^72^, SRR18500466^73^, SRR18500478^74^, SRR18500489^75^, SRR18500490^76^ and SRP151715^77^. The others RNA-seq data have been deposited in the NCBI SRA database under the SRR16371984^78^, SRR16371985^79^, SRR16371986^80^, SRR16371987^81^, SRR16371988^82^, SRR16371989^83^, SRR16371990^84^, SRR16371991^85^, SRR16371992^86^, SRR16371993^87^, SRR16371994^88^, SRR16371995^89^, SRR16371996^90^, SRR16371997^91^ and SRR16371997^92^, which is associated with the Bioproject ID PRJNA771936. The assembled genome sequence has been deposited at GenBank with accession number JAQSGO000000000^93^. Other data, such as gene structure annotation, predicted CDS and protein sequences, annotation of TEs, tandem repeat sequences, tRNA genes, miRNA genes, snRNA genes, and rRNA genes, are available at FigShare database^94^.

## Technical Validation

Assessment of the completeness of the genome assembly using CEGMA indicated a 95.56% (Haplotype: DkaA 89.92%; DkaB 90.73%; DkaC 91.13%; DkaD 86.69%; DkaE 89.11%; DkaF 86.69%) coverage of the conserved core eukaryotic genes, while the BUSCO (v5.2.2; embryophyta odb10 database)^95^ results indicated that the genome and gene set was 99.50% (Haplotype: DkaA 92.70%; DkaB 93.50%; DkaC 92.10%; DkaD 90.10%; DkaE 90.20%; DkaF 84.60%) and 97.50 (Haplotype: DkaA 87.10%; DkaB86.60%; DkaC 86.10%; DkaD 83.90%; DkaE 84.60%; DkaF 79.80%) complete, respectively (Tables 11, 12), showing that the individual haplotypes lack genes present elsewhere in the genome. Additionally, 99.86% (Haplotype: DkaA 94.88%; DkaB 94.59%; DkaC 93.70%; DkaD 93.94%; DkaE 92.97%; DkaF 90.09%) of the high-quality short reads were mapped back to the assembly (Table 13). All in all, these results of these assessments indicate to us that the *D. kaki* genome assembly is complete and high quality.

**Table 11:** Assessment of the completeness of the genome assembly.

Genome	BUSCO	CEGMA
The whole genome	C:99.50% [S:0.90%, D:98.60%], F:0.20%, M:0.30%, n:1614	95.56%
DkaA	C:92.70% [S:89.70%, D:3.00%], F:2.00%, M:5.30%, n:1614	89.92%
DkaB	C:93.50% [S:91.00%, D:2.50%], F:1.70%, M:4.80%, n:1614	90.73%
DkaC	C:92.10% [S:89.70%, D:2.40%], F:1.70%, M:6.20%, n:1614	91.13%
DkaD	C:90.10% [S:87.00%, D:3.10%], F:2.00%, M:7.90%, n:1614	86.69%
DkaE	C:90.20% [S:87.60%, D:2.60%], F:2.70%, M:7.10%, n:1614	89.11%
DkaF	C:84.60% [S:82.20%, D:2.40%], F:2.10%, M:13.30%, n:1614	86.69%

**Table 12:** Assessment of the completeness of the gene set.

Gene set	BUSCO
The whole gene set	C:97.50% [S:2.50%, D:95.00%], F:1.90%, M:0.60%, n:1614
DkaA	C:87.10% [S:83.50%, D:3.60%], F:5.30%, M:7.60%, n:1614
DkaB	C:86.60% [S:83.10%, D:3.50%], F:6.20%, M:7.20%, n:1614
DkaC	C:86.10% [S:82.90%, D:3.20%], F:6.60%, M:7.30%, n:1614
DkaD	C:83.90% [S:79.70%, D:4.20%], F:6.50%, M:9.60%, n:1614
DkaE	C:84.60% [S:81.70%, D:2.90%], F:6.60%, M:8.80%, n:1614
DkaF	C:79.80% [S:76.10%, D:3.70%], F:5.70%, M:14.50%, n:1614

**Table 13:** Coverage statistics of *D. kaki* genome.

The whole genome	Reads	Mapping rate (%)	99.86
		Average sequencing depth	27.29X
		Coverage (%)	99.72
	Genome	Coverage at least 4X (%)	99.45
		Coverage at least 10X (%)	97.2
DkaA	Reads	Mapping rate (%)	94.88
		Average sequencing depth	306.75X
		Coverage (%)	99.85
	Genome	Coverage at least 4X (%)	99.79
		Coverage at least 10X (%)	99.73
DkaB	Reads	Mapping rate (%)	94.59
		Average sequencing depth	325.76X
		Coverage (%)	99.93
	Genome	Coverage at least 4X (%)	99.89
		Coverage at least 10X (%)	99.84
DkaC	Reads	Mapping rate (%)	93.7
		Average sequencing depth	341.1X
		Coverage (%)	99.97
	Genome	Coverage at least 4X (%)	99.95
		Coverage at least 10X (%)	99.93
DkaD	Reads	Mapping rate (%)	93.34
		Average sequencing depth	346.92X
		Coverage (%)	99.99
	Genome	Coverage at least 4X (%)	99.97
		Coverage at least 10X (%)	99.95
DkaE	Reads	Mapping rate (%)	92.97
		Average sequencing depth	356.66X
		Coverage (%)	99.99
	Genome	Coverage at least 4X (%)	99.98
		Coverage at least 10X (%)	99.96
DkaF	Reads	Mapping rate (%)	90.09
		Average sequencing depth	375.72X
		Coverage (%)	99.99
	Genome	Coverage at least 4X (%)	99.99
		Coverage at least 10X (%)	99.97

**Fig. 5:**
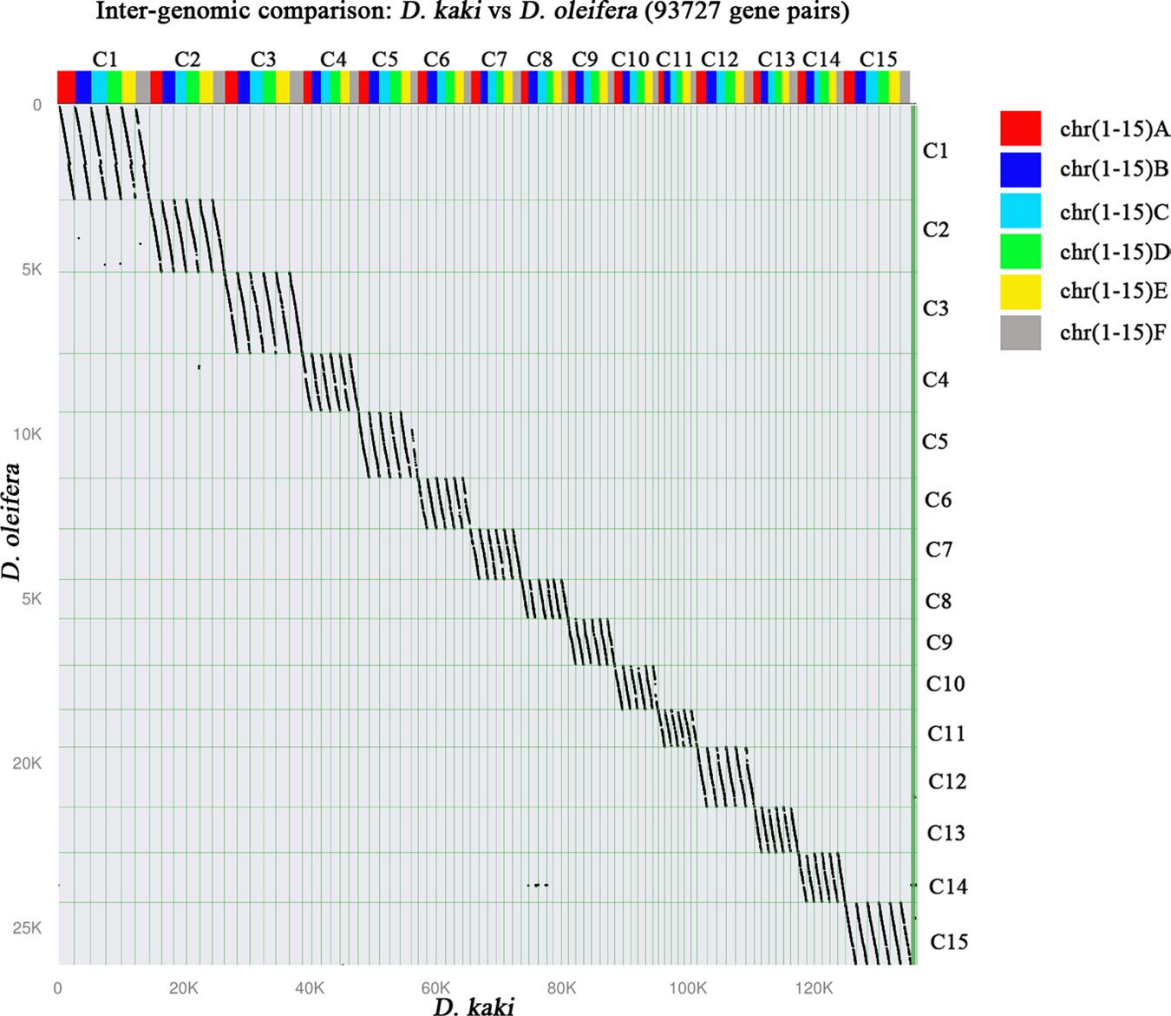
The syntenic dot plots of *D. kaki* and *D. oleifera*.

Inter-genomic comparison analysis revealed a distinct 6-to-1 syntenic relationship between *D. kaki* and *D. oleifera* (Fig. 5), which further supported the high quality of the *D. kaki* assembly.

## Data Availability

All software used in this work are in the public domain, with parameters described in the Methods section. The commands used in the processing were all executed according to the manuals and protocols of the corresponding bioinformatics software.
